# The dynamics of musical participation

**DOI:** 10.1177/1029864920988319

**Published:** 2021-03-19

**Authors:** Andrea Schiavio, Pieter-Jan Maes, Dylan van der Schyff

**Affiliations:** Centre for Systematic Musicology, University of Graz, Austria; IPEM, Department of Art, Music, and Theatre Sciences, Ghent University, Belgium; Melbourne Conservatorium of Music, The University of Melbourne, Australia

**Keywords:** Musical participation, interaction, dynamical systems theory, music performance, social cognition

## Abstract

In this paper we argue that our comprehension of musical participation—the complex network of interactive dynamics involved in collaborative musical experience—can benefit from an analysis inspired by the existing frameworks of *dynamical systems theory* and *coordination dynamics*. These approaches can offer novel theoretical tools to help music researchers describe a number of central aspects of joint musical experience in greater detail, such as prediction, adaptivity, social cohesion, reciprocity, and reward. While most musicians involved in collective forms of musicking already have some familiarity with these terms and their associated experiences, we currently lack an analytical vocabulary to approach them in a more targeted way. To fill this gap, we adopt insights from these frameworks to suggest that musical participation may be advantageously characterized as an open, non-equilibrium, dynamical system. In particular, we suggest that research informed by dynamical systems theory might stimulate new interdisciplinary scholarship at the crossroads of musicology, psychology, philosophy, and cognitive (neuro)science, pointing toward new understandings of the core features of musical participation.

In their many cultural and historical manifestations, musical practices often involve collaborative and participatory behaviors (Small, 1999; Turino, 2008). These practices include forms of performance, musical learning and listening, improvisation, and (collaborative) composition that play out in diverse contexts: live concerts, religious ceremonies, recording sessions, DJ mixsets, educational institutions, informal settings, therapeutic environments, and more. Recent research that explores these complex forms of interpersonal activity typically makes use of the empirical and theoretical tools of social psychology (see e.g., Hargreaves & North, 1997), and embodied cognitive science (see e.g., Lesaffre et al., 2017; Moran, 2014), among other domains. Much work in the field investigates various aspects of real-time interaction between participants, including sensorimotor synchronization (Repp, 2005), coordination (Zamm et al., 2015), prediction (Heggli et al., 2019; Vuust & Witek, 2014), and the ways in which synchrony increases pro-social behavior (Stupacher et al., 2017). Different methodologies and theories have been advanced to cross-classify the dynamics and main properties that characterize musical participation. We suggest, therefore, that a common analytical vocabulary might be useful to help overcome (or, at least, reduce the distances between) the terminological, conceptual, and methodological discontinuities produced by different approaches.

We thus examine below how the existing frameworks of *dynamical systems theory* (DST) (e.g., Katok & Hasselblatt, 1999; Padulo & Arbib, 1974), and *coordination dynamics* (e.g., Kelso, 1992, 1994, 2001) can offer a novel set of conceptual tools to help us understand and describe the dynamics of musical participation in greater detail. We begin by considering recent work in skill acquisition, group dynamics, music education, and cognitive science that already moves in this direction, particularly contributions that highlight the social, and pedagogical advantages of taking a relational, creative, and collaborative orientation toward musical activity. Next, we introduce relevant concepts from DST and coordination dynamics. These concepts are used to illustrate a number of the central features of joint musical experience such as prediction, adaptivity, social cohesion, reciprocity, and reward. While most musicians involved in collective forms of musicking have some familiarity with these terms, and their associated experiences, we currently lack an analytical vocabulary to target them more precisely. In addressing this limitation, we suggest that the complex phenomenon of musical participation should be characterized as an open, non-equilibrium, dynamical system. To conclude, we consider how research informed by DST might stimulate new interdisciplinary scholarship at the crossroads of musicology, psychology, philosophy, and cognitive (neuro)science; and how it could therefore point toward new understandings of the core features of musical participation, moving from descriptions to explanations.

## Social Dynamics of Skill Acquisition and Performance

In research on the social dynamics of small and large musical ensembles (e.g., D’Ausilio et al., 2012; Glowinski et al., 2012; Luck & Toivanenen, 2006; Murnighan & Conlon, 1991; Rasch, 1988), interactive musical agents (i.e., performers) are often conceived of as individual units embedded within a larger system (see D’Ausilio et al., 2015; Volpe et al., 2016). The emergence of collaborative roles and leadership within these multi-agent systems have been investigated systematically (Davidson & Good, 2002; King, 2006). For example, Timmers and colleagues (2014) note that while temporal information in a small ensemble may be coordinated by a leader who is responsible for indicating tempo and phrasing through bodily and sonic cues, the role of the other musicians is not simply passive: they can still actively participate, respond, and compensate for discrepancies (see Keller, 2001; Schiavio & Høffding, 2015). A challenge for this research is presented by improvising musicians (Bailey, 1993; Berliner, 1994; Borgo, 2007; Sudnow, 1978). Jazz and free improvisers collaboratively create new musical patterns in real-time, where the role of leader is not always strictly defined. More recent experimental work by Walton and co-workers (2015) has explored the emergent patterns of bodily coordination that musicians develop in joint improvisation, highlighting how forms of motor coherence emerge between the interacting agents at multiple time scales, and how this is guided by mutually modulatory self-organizing dynamics across corporeal and sonic dimensions (see also van der Schyff et al., 2018). This entails the negotiation of sensorimotor co-regulation between the performers, which enables the constitution of an evolving musical environment, and that, in turn, shapes the patterns of movement and sound that are enacted as the musical interaction unfolds.

Recent work along similar lines on skill acquisition in music, dance, and sport, has adopted conceptual tools from phenomenological philosophy and cognitive science to describe how individuals can shape each other’s learning (e.g., Schiavio et al., 2019a). Here, co-participation is seen as one important driver of skill acquisition, as it requires individuals and groups to negotiate different emotional, social, and behavioral configurations in real-time. It is argued that these types of collaborative activity, when directed toward skill acquisition, can aid novices in exploring and developing their performative potential—for example by adapting to novel musical challenges from peers—and that they can also help more advanced individuals to optimize their skills in lived, practical contexts (see Gesbert & Durny, 2017; Schiavio et al., 2020). These participatory forms of learning necessarily involve a considerable amount of uncertainty; for example, musicians are required to adjust to real-time shifts and perturbations, and they must coordinate their actions to keep the parameters of musical performance intact. As such, contextual adaptivity also plays an important role in the development of novel and creative skills (see Sawyer, 2003, 2006, 2007; van der Schyff, 2019). Consider, for example, how pick-up basketball games, free improvisations and other forms of joint music making (e.g., collaborative composition) do not always adhere to rigid rules or predetermined outcomes. Instead, settings and rules are often developed via group negotiations, explicit or tacit, that are based on the precise context of the game or music making activity. These give rise to novel behavioral, emotional, and/or social configurations that may display new and adaptive functionality. In other words, these kinds of collaborative activities and performances involve forms of creative self-organization that evolve over time according to moment-to-moment contingencies. This can lead to shifts in their predicted outcomes, as well as rapid behavioral adaptations and the development of compensatory actions and configurations (see Davis et al., 2015; Gruber, 1989; Hristovski et al., 2011, 2012; Kimmel et al., 2018). The following two examples may clarify this point.

If two teams form to play a game of street basketball, and one team is clearly less motivated than the other to finish the game because of their lack of athleticism, members of the opposing team can arrange novel settings to make up for it and keep the game interesting. For instance, they can modify the duration of the game, making it shorter than usual, by agreeing that the winner would be the first team to score five baskets instead of ten. By displaying a good balance between novelty and functionality, this new format can keep the game competitive and enjoyable for all parties involved. Similarly, imagine two flamenco guitarists giving a street performance. By modulating the intensity of their *rasgueado* (the right-hand strumming that is the signature of flamenco) they can affect the ways listeners actively engage with the music. For example, shifts in the musical environment can influence the movements of dancers. However, each dancer will have different levels of expertise, and different techniques and styles; thus, each dancer will respond to the music in their own way. The ability to negotiate the rhythmical nuances capable of facilitating the participation of others requires impressive control as well as the capacity to generate, and adapt to, novel, unpredicted, musical contingencies (e.g., the interaction between the guitarists, and between the guitarists and the dancers) without weakening the intensity of the performance. This not only involves the redeployment of existing musical patterns and configurations known by the players, but also the adaptation of those patterns to the context, and the co-construction of novel forms and interactions to deal with unpredicted factors in the unfolding musical environment. In these two examples from sport and music, the rules and dynamics of the participatory activity are negotiated and co-developed to both maintain stability and to push the dynamics of the performance in new directions, that is, to keep the performance interesting, challenging, and coherent. Without these pragmatic abilities to enact new forms of group cohesion, coherent joint musical activity is arguably not possible.

These types of abilities entail a continuous flow of (motor) information— the tightly coupled loops of action and perception that guide joint performative activity—that cannot be easily separated from the context in which they are situated (Kimmel & Rogler, 2018; Kimmel et al., 2015, 2018). Rather, action, perception, and environmental conditions co-evolve via mutually modulatory dynamics; as one factor changes, so does the other. Accordingly, the emergence and development of the real-time patterns of interpersonal coordination developed by co-improvisers, or other performers working in participatory contexts, cannot be fully captured by analyses of how individuals *respond* to each other or to their environment. Indeed, in musical settings, the full spectrum of joint coordination emerges and evolves in the interaction *between* musicians and their contingent milieu. This means that a multi-agent level of analysis is necessary to describe the unfolding dynamics of such contexts—a level of analysis that embraces collectivity, reciprocity, and ecological contingencies, and which goes beyond the sole focus on the individual agent (see Cummins, 2018; Fuchs, 2018; Fuchs & De Jaegher, 2009).

## Insights from Music Education

As we have seen, the dynamics of joint musical performance depend on the moment-to-moment relationships between the players involved (see Moran, 2017; Ryan & Schiavio, 2019), which entail a constant push and pull between stability and instability. Sometimes this means dealing collectively with factors that might threaten the coherence of the musical environment, by adapting, for example, to a rhythmic or pitch error, making a tuning adjustment, or shifting the interpersonal coordination associated with a musical groove. Indeed, collaborating musicians need to be able to resist and/or adapt to a range of perturbations, but they also need to remain open and highly sensitive to the musical environment being created through performance, so that they can perceive, and participate in, the development of novel musical possibilities (Torrance & Schumann, 2018). Consider how, in a jazz improvising context, a soloist might introduce an unexpected rhythmic, melodic, or harmonic shift (sometimes all at once). The accompanying musicians will adapt to this creatively, and in turn influence the development of the music. Additionally, the constantly evolving nature of joint musical activity is such that these interpersonal dynamics are rarely, if ever, symmetrical, as musical agents rely on each other to perform various tasks (e.g., cueing, tuning, rhythm, tempo and phrasing, dynamics and more), with different players taking on adaptive roles in a variety of ways as the music unfolds.

These kinds of interpersonal dynamics are of growing interest in the context of music education (e.g., Biasutti & Concina, 2018; Borgo, 2007; Nielsen et al., 2018; Simones, 2019). As most learning unfolds in interaction, for example with teacher(s) and peer(s), this area represents an ideal domain in which to investigate how musical participation develops in a variety of social groupings. These include dyads (e.g., teacher–student, student–student, and teacher–teacher) as well as more asymmetrical sets of participants (e.g., one teacher with many students). Notably, there is now a growing interest in studying how musical learning happens in informal contexts, and how this might aid educators in revising current pedagogical approaches. In such contexts, musical learning is not guided by an externally prescribed agenda, but develops directly from the shared activities of the musicians themselves, who acquire their musical skills as they collaboratively explore possibilities, find and solve musical problems, create new music, and improvise together (Gaunt & Westerlund, 2013; Green, 2008).

Because musical learning involves such an impressive range of styles, experiences, approaches, methods, and forms, it might be useful to conceptualize it as a network of interacting trajectories that evolves over time. On this view, novel patterns of musical action can be seen as arising (or, in this case, self-organizing) from the reciprocal interplay of the agents who comprise the network, rather than only through the linear process of acquiring and elaborating on external or pre-given knowledge. This leads to the argument that the rich variety of responses, experiences, and musical outcomes that develop as joint musical learning takes place cannot be explained only in terms of the acquisition and reproduction of facts and techniques that are already established. Rather, it involves the engaged co-enaction of musical understanding through praxis (Elliott & Silverman, 2015). Current research on informal learning contexts highlights the potentially transformative nature of the relationship between agents and shows how these processes can play a decisive role in the development of musical skills by affecting factors such as motivation, emotion, confidence, creativity, cohesion, and trust. In all, it is argued that there is much more to musical learning than the repetition of movements during practice, and the internal elaboration of knowledge leading to representation of goals and motor schemas (see e.g., Schiavio & van der Schyff, 2018). Although these remain important aspects of musical training (see Lehmann, 1997), the ways in which motor, social, and emotional factors combine to link students, instruments, and teachers at multiple levels are also major aspects of their learning experience, which may require different explanatory tools.

Attempts to meet this challenge often involve rethinking the aims and nature of music making more generally, both theoretically and practically. Accordingly, researchers have reconsidered traditional assumptions about the role of information and its unidirectional transmission from teacher to students, to explore in more detail the personal, cultural, social, and pedagogical value of musical practices based in improvisation, collaboration, and creativity (Bailey, 1993; Borgo, 2007; Sawyer, 2007; van der Schyff et al., 2016). This moves the focus away from the *internal* processing of *external* knowledge to embrace a perspective that emphasizes the learner’s ability to participate in and transform the musical environment they inhabit, sidestepping the dichotomy between objective knowledge and its subjective elaboration (see Schiavio, 2019). Similarly, studies of peer-to-peer learning, community music, and collective pedagogies inspire an important shift in theory and practice. For example, recent work on peer interaction in musical learning places an important focus on the connection between students’ motivation and creativity, and their sense of belonging to the group (Kototsaki & Hallam, 2007, 2011; see also Johansen & Nielsen, 2019). It has also been argued that fostering safe and positive environments encourages students to take on more responsibility and develop their own musical identities, highlighting once more the recursive co-determination of social, cultural, emotional, and motivational factors (e.g., Schiavio et al., 2019b). As noted by Ilari and colleagues (2016), such lines of enquiry reflect a new emphasis in scholarly research on social and cultural aspects of learning.

In summary, the traditional model of musical learning, based on the unidirectional transfer of knowledge from expert to novice, is being replaced by a range of new relational perspectives on musical development. And where previous contributions were more interested in specific musical outcomes based on relationships between age, quantity of practice, and the development of skills, and how these relate to the ability to perform in certain prescribed contexts (e.g., the performance of notated music in specific genres or styles, which might be considered merely reproductive), these new perspectives highlight the potential for music research to seek a better understanding of many aspects of social interaction across cultures, social contexts, and skill levels. As mentioned above, these developments in the field demand new theoretical models and explanatory tools to describe the complex layers of meaning making involved in joint musical practices. Here, recent contributions from embodied music cognition have started to offer a way forward (Demos et al., 2014; Laroche & Kaddouch, 2015; Leman, 2007; Maes et al., 2014). These contributions explore musical participation as a property of the relationship between bodies-in-action and the sonic environments they generate; the agents involved in the musical event become constitutive parts of the complex network of reciprocal interactions that shapes, and is shaped by, the musical event being enacted (Loaiza, 2016; Schiavio & De Jaegher, 2017; van der Schyff et al., 2018). In line with this, we now consider recent work in embodied cognitive science, which sheds new light on the idea of *participation*. This will allow us to present some new possibilities for thinking about musical participation in the context of education and performance.

## Participation: Rethinking the Level of Analysis

A good way to begin thinking about musical participation is to start with more general situations in which people collaborate and interact meaningfully in their everyday life. To drive a car, move through a shopping mall, communicate with a bank teller, perform music with others, and carry out the myriad of other shared activities necessary to achieve the goals that characterize daily life, a person needs to integrate their knowledge about the other people involved within a set of shared understandings that guide conduct within specific contexts; our capacity to comprehend and engage meaningfully with other agents depends on cultural norms and social habits that are often so ingrained in us that we take them for granted. However, in addition to these broader socio-cultural factors, the ability to participate in social environments needs to be understood also in terms of the complex mental operations that allow us to make sense of actions and goals of other people, to comprehend their behaviors, and anticipate their intentions.

A number of researchers have approached this problem by arguing that these mental operations play out as inner representations of what is occurring in the social world and in the minds of other people: our capacity to understand others, according to this view, is thus rooted in the ability to conceptualize the minds of those we engage with (see e.g., Goldman, 2006). To the degree that different agents can come to functionally similar understandings of a given situation—and are therefore able to integrate the actions of others into their own motor plans^1^—they are said to be in possession of *shared representations* (see e.g., Pezzulo, 2011). Put simply, this view has traditionally been used to explain successful participatory action and the realization of common goals (e.g., driving a car in traffic, performing in a musical ensemble, and so on). Developments of this perspective increasingly recognize the importance of body and action in shaping empathy and sociality. Indeed, it has been suggested that in perceiving the goal-directed movements of others, we engage neural mechanisms involved with producing such movements ourselves, giving rise to internal *simulations* of what others might feel or do (see Gallese, 2007, 2014). This orientation is often interpreted as conceptualizing social understanding as a property of individuals who use sensory information to represent or simulate external matters.

While this approach offers valuable explanatory possibilities, it also has some shortcomings. For instance, it remains unclear how—once such forms of mind-reading or simulation processes are accomplished—a person might (often instantaneously) select the appropriate behavioral responses from their motor repertoire^2^ to complement the actions of the other agents successfully and thereby carry out the joint task (see Sebanz et al., 2006). It is also unclear how body-based (social) knowledge arises. If research focuses on individuals’ internal simulation processes only, the genuinely subjective, personal experiences of social interaction and participation, and the developmental processes that underlie them, may remain underdeveloped (see e.g., Fuchs & De Jaegher, 2009). Experience tells us that in interacting with others we learn contextually relevant skills that are both repeatable and adaptable to the contingencies of the moment. Yet we can only develop this kind of understanding by reference to our lived histories of interaction within social and material environments; we learn and develop *with others*, and mind and experience are fundamentally and essentially *socially embodied.* This has led some to argue that while simulation processes might be an important aspect of social understanding, they cannot fully explain empathy (Gallagher, 2012).

In response to these kinds of concerns, many researchers have begun to move in a different direction. In fields spanning social cognition, psychology, and neuroscience, for example, novel theoretical and empirical work is emerging that places considerable focus on a multi-agent approach that more closely engages with the experiential, meaningful, and transformative aspects of reciprocal interaction, corporeality, and participation (De Jaegher, 2007; De Jaegher & Di Paolo, 2009; Gallagher, 2007, 2008; McGann, 2014). As Cummins (2014) puts it, “social cognitive neuroscience has recently begun to recognize that nervous systems of interacting individuals behave quite differently from those of solitary subjects, and often become inter-dependent.” Indeed, where previous models (e.g., those committed to simulation and representation) tended to focus on describing the processing that takes place within the brains of individuals, more recent work examines social cognition in terms of complex multi-layered networks that include bodily, neural, and environmental trajectories in the course of real-time interaction (Genvis et al., 2012; Hasson et al., 2012; Tognoli et al., 2011). In other words, social participation is now explored as a synergistic phenomenon that depends as much on environmental and corporeal dynamics as it does on the activity of the brain.^3^

Notably, these new approaches to social cognition include the *interactive brain hypothesis* (Di Paolo & De Jaegher, 2012; De Jaegher et al. 2016), and the *second-person approach* to neuroscience (Schilbach et al., 2013). The former refers to an account inspired by embodied and enactive cognitive science (Thompson, 2007), which aims to shed new light upon the relationship between neural processes and social behaviors (see Gallagher et al., 2013). The main idea is that a brain in isolation cannot reveal the properties, mechanisms, and dynamics of (social) cognition. According to this view, the brain, the body, and the world comprise a system that enables social interaction to occur—a nexus of neural-corporeal-environmental interactivities that evolves with and within the social dynamics being enacted. If one component were to be removed from the system, the entire nexus—or network—would collapse. It is then not enough to focus on one component alone (i.e., the brain) when exploring the evolution of the brain-body-world system. This also aligns with the second-person approach to neuroscience mentioned above, which seeks to reformulate the problem of other minds (i.e., how do we really know that others possess minds functionally similar to ours?) in terms of embodied experience and social participation, including emotional-empathic engagement. Here it is argued that the embodied dynamics of participatory activity can help capture fundamental aspects of social cognition as they highlight the importance of reciprocal experiences of “moving, gesturing, and engaging with the expressive bodies of others” (Gallagher, 2017, p. 20), and show how these interpersonal engagements lead to the enactment of patterns of movements and feeling whose significances are shared between agents (for similar insights derived from research on music making, see Godøy, 2015; Krueger, 2013, 2014; Leman, 2007; Maes, 2016; Moran, 2014).

At the heart of these approaches, then, is a recognition of the fundamental role of experience and bodily interaction within a brain-body-world milieu. Accordingly, the domain of participation becomes part of the *extended system* in which interaction unfolds. This trades the focus on individuality for a focus on multiple agents producing a narrative in which socially emergent properties can be considered in detail. What experiential, emotional, and behavioral factors are shared between the people who are interacting with one another (*interactors*)? In what sense does reciprocal interaction influence and extend individual sense making and musical experience? And how can we best describe the ongoing, live, entanglement that permeates social musicking? In the next section, we engage with such questions from a perspective that explores the transitory, fluctuating, and shared dynamics involved in musical interaction.

## Musical Participation as an Open, Non-equilibrium, Dynamical System

From the perspective introduced above, musical participation does not necessarily involve linear stimulus-response schema based on perceptual inputs and behavioral outputs. This is because the factors that determine shifts in the global state of the system (music and interactors) are already structurally and functionally integrated in the system itself. In other words, the social musical system operates in a synergistic fashion whereby the components of the network constantly influence each other. This means that the evolving trajectory of the system (i.e., the realization of the musical event over time) is always open to a number of possible (e.g., kinesthetic, metabolic, affective) shifting adaptations that characterize each interactor. Importantly, the modulatory activity of the musical system’s structural dynamics leads to a dense set of causal interactivities that transform the musical event recursively through continuous feedback and feed-forward processes (see Fogel, 1992, 1993). This “continuous reciprocal causation” (Clark, 1997, p. 165) produces, among other things, the real-time negotiation and co-regulation of emotional and metabolic states (Colombetti, 2014) in which non-verbal forms of communication play a key role in facilitating the success of the interaction.

Coordination dynamics and the mathematical tools of DST are well suited to address the complex dynamics of musical participation we began to discuss above. They offer a rich vocabulary that can help us describe important aspects of musical participation (e.g., those related to coordinated behavior and their underlying mechanisms) in a precise manner, providing music scholars and performing artists with new tools to explore the experience of joint musicking and their underlying structuring laws more effectively. The framework of coordination dynamics is understood as “a line of scientific inquiry that aims to understand, through theory, analysis, and experiment, how patterns of coordinated behavior emerge, persist, adapt and change in living things in general and human beings (and brains) in particular” (Kelso, 2003, p. 45). DST involves a set of mathematical tools based on differential equations for describing the behavior—which varies over different time courses—of systems comprised of multiple mutually influencing components. Because such systems are in a continuous state of flux, with a number of variables involved in their evolution, they are described as non-equilibrium systems. This description contrasts with that of equilibrium systems, which exhibit balanced dynamics and therefore remain static in terms of their temporal development. Importantly, while non-equilibrium systems change over time, they nevertheless exhibit patterns in their development (e.g., weather formations, bird flocking, and insect swarming) that are known as *emergent properties*. Furthermore, they are often described as *open* because they can both influence and be influenced by other factors. Living systems are prime examples of these kinds of open, non-equilibrium systems, as they adapt to and shape environmental conditions in a variety of ways.

In what follows we bring together several insights from these approaches to describe musical participation as an *open, non-equilibrium, dynamical system*. To do so, we deliberately use concepts deriving from both coordination dynamics and DST loosely and more informally than they do, aiming to make relevant parallels and comparisons with musical activities. For example, when discussing DST, we do not use mathematical models or equations, but rather consider how its key insights could help guide future research from a theoretical perspective. While this informal strategy is limited to a certain extent, it nevertheless allows us to introduce the conceptual tools associated with these approaches in ways that are intended to be accessible to a broad range of music researchers interested in multi-causality. That said, it is also possible that future music research could make good use of the more empirically rigorous aspects of these approaches (for some preliminary examples see Borgo, 2005; Walton et al., 2015, 2018).

A basic characteristic of an open non-equilibrium dynamical system is that it is composed of various interrelated components, or subsystems, which interact with each other. In the case of human music interaction, the basic components are constituted by (embodied) agents, their musical instruments, and their environment, each composed of many more coupled and integrated sub-components. Again, such systems are considered open when an exchange of energy, matter, and information occurs among their components and between the system and its surroundings. Because of these exchanges, open systems may exist in a number of different *states*, which are realized, importantly, by nonlinear relationships between the system’s individual components and the environment in which the network operates, rather than by the behavior of its individual components. Again, a change in one area of the system entails a shift in the other areas of the system as its components behave in a mutually modulatory fashion. For example, states of balance occur when there is constancy in the system’s relationships (Recordati & Bellini, 2004). An open system is never fully equilibrated (which is why it is known as a non-equilibrium system), and it relies on the continuous exchanges of components and information to maintain its structure and functionality (homeostasis); alternatively, it can be triggered by these exchanges to start evolving qualitatively new forms of organization and behavior. The evolution of the different states and configurations of the system can be captured mathematically, at least in part, using the tools of DST (see also Favela, 2020a, 2020b).

Conceiving of musical interaction in such terms could provide valuable theoretical and methodological tools for capturing the complexities of musical participation, as well as its underlying behavioral and neural principles. Traditionally, the recurrent loops of reciprocal causation between elements internal and external to the system have often been thought of as resulting from a series of stimuli provided by one or more people and responses by others, who would achieve the desired musical outcome by modulating their feedback to each other. In the context of Western classical music, for example, consider how duets are usually conceived of as dialogs between individual agents who produce musical ideas and convey them to each other as the performance unfolds. As Goodman puts it, in ensemble situations

each performer continually listens to the expressive nuances of sound emanating from fellow performers, such as fluctuations in timing, gradations in dynamics, and changes in articulation, timbre and intonation. In effect, the individual’s concentration is divided between monitoring the sound produced from his or her own part and attending to the sound produced from the rest of the group.(Goodman, 2002, p. 156)

This characterization provides a good starting point for describing how musical parameters and individual experiences are developed and exchanged in the process of musical interaction. However, other aspects also need to be addressed: the reciprocity of musical actions, the co-evolving nuances of mutual interactivity, the feeling of being together with others, and the role of co-presence (see Himberg et al., 2018).

Suppose you were playing with a famous rock band, live, in a stadium. It would not be possible for the outcome, emerging from a complex web of interactivities involving all the members of the band, to be fully realized without a supportive technical team to take care of the mixers, the amplifiers, the tunings, the visual effects on the stage, and so on. The crew and technicians would also play a crucial role in enacting the dynamics of the musical performance. To add yet another degree of complexity, it can be argued that members of the audience also take part in realizing the performance (see Moelants, et al., 2012; Schaerlaeken et al., 2017). During a live show, their role could be observed if the performers were deliberately to involve the audience in shaping the performance, for example by encouraging them to join in with the chorus of a song. And more generally, the presence and responsiveness of the audience influences the emotional and motivational states of the performers, whose actions in turn affect the audience, and so on. Thus, the participatory system designed to create an optimal performance consists of the band, the crew, and the audience, in which all components make contributions—in different ways and to different degrees—to achieve the kind of dynamics that result in appropriate balances of stability and perturbation to produce a show that is both coherent and exciting. Of course, this is not an easy task. Even experienced performers cannot accommodate all the unforeseen situations they may encounter while on stage: electronic devices may malfunction; a singer may develop a sore throat and be unable to perform adequately; the audience may react badly to an improvised solo. And even when performances go well, constant adaptations are required to maintain their flow.

It can thus be seen that the musical system described here is always co-determined by ecological factors, as participants actively incorporate elements of their own personal histories, moment-to-moment subjective states and intentions, and physical and socio-cultural environment; performance sustains itself by transforming different variables over time. Rather than focusing on causal, linear chains of events affecting each element of the network one after the other, we can tell a more complex story in which all the sub-components of the system, and therefore the whole system, are fluctuating as the result of mutually modulating feedback and feed-forward effects (see Recordati & Bellini, 2004). This reinforces the view that open systems are never fully in equilibrium. Their states continuously reorganize themselves in light of the recursive interactions of its components, which can either work to maintain and optimize their current structure and functionality, or generate qualitatively new forms of organization and behavior. Importantly, the reorganization of states involves not only macro-changes, at the level of kinematics or musical outcomes, but often more subtle micro-phenomena deriving from changes of agency, or in the performers’ emotions, awareness, and so on. These shifts lead to transformations that may be rapid and subtle, but nevertheless shape skilled interaction and expression, as performed by the musician and experienced by the audience.

Suppose again that you were a member of the rock band mentioned above. This time imagine you were playing the keyboards in the pre-concert rehearsal and you felt that, just after the introduction to one song, the drummer had begun to play faster. At first, the rhythm guitarist and bass player might not notice the increase in tempo, as they would be able to maintain the rhythm of the verse and pre-chorus with some consistency. When the main chorus began, however, you would all realize that something was wrong and that you would need to find a solution before the first entry of the singer. In such a situation, no member of the band would be able to rely on another member’s initial impulse to drive the necessary recalibration; each member would have to act quickly, given the collective constraints imposed by the task. In this case we can see the dynamics of the system rapidly evolving, in such a way that instability would threaten the continuation of the music. To bring the system back within sustainable parameters would require an adaptation on the part of all the performers, an ability essential to skillful music making.

Conceiving of joint musicking as an open, non-equilibrium system offers a way to describe the situation encountered by the members of the rock band in the light of the principles of *self-organization*. Indeed, the various contingencies and perturbations any band might encounter while playing together are not best described by a single factor that triggers a chain of responses and subsequent adaptations: the states of a system and its dynamics may be better understood, rather, by exploring the sets of *relationships* that exist between the system’s components. These relationships can be measured as variables, known as *order parameters* or *collective variables*. In coordinated rhythmic movement, an important order parameter is the *relative phase*, which captures the temporal relationships in the co-regulated timing of two or more body parts, or people in the case of interpersonal interaction (Haken & Portugali, 2016; Kelso, 1995). An essential idea here is that the interaction between components (be they body parts or people) is driven toward specific *states* of a collective variable, so called *attractor states*. In the case of the collective variable relative phase, these attractor states correspond to in-phase and anti-phase temporal relationships (as illustrated by the Haken-Kelso-Bunz model) (Haken et al., 1985). For example, as musicians enact the recurrent patterns of bodily action required to maintain a groove, they align their movements such that the sounds they produce occur as relationships that are in phase with the pulse and meter of the shared musical environment. Maintaining these balances requires constant adaptations at both micro level (e.g., how the fingers interact with the strings of the bass) and macro level (e.g., how bandmates interact between themeselves and with the audience).

It is also interesting to note that, in joint musical activities, there is a more general preference, or attraction, toward small integer ratios in both temporal and melodic-harmonic relationships (e.g., duple meter as opposed to complex polyrhythm), as demonstrated in the bodily rhythms of people engaged with music (see Coorevits et al., 2017; Jacoby & McDermott, 2017; Ravignani et al., 2016, 2018), as well as in the use of particular tone scales in the musical repertoire across cultures (Bigand et al., 2014; Bowling & Purves, 2015; Gill & Purves, 2009). However, research has shown that, with practice and experience, agents can perform and maintain complex polymetric relationships that exhibit attractor states that are as stable as simpler ones (Amazeen et al., 1996).

In any case, because components constrain and complement each other locally to ensure that the musical system is directed toward specific attractor states (maintaining rhythmic patterns and performing within melodic and harmonic constraints), the system can be understood as *self-organizing*. The multi-component, recursive, interactive, and adaptive nature of self-organizing processes means that systems can enact and re-enact attractor states that are similar, but that also exhibit certain degrees of flexibility and freedom within constraints. These dynamics can help to describe how an ensemble can work coherently within the rhythmic, harmonic, and melodic constraints that define a style—or that afford various performances of a piece—while also exploring and adapting to various novel behavioral patterns that emerge from each performance.

## Nonlinear Methods for the Study of Musical Participation

The conceptual and mathematical resources of DST have led to novel insights into the behavioral organization of various complex systems and their functional patterns of reciprocal causation, beginning with the fundamental interactions between the basic components of the system (Beer, 2000; Strogatz, 1994, 2001; Thelen & Smith, 1994). Accordingly, we argue that this approach may provide a richer understanding of the complexities, diversities, and dynamics inherent in musical participation. That said, studying musical participation from a nonlinear dynamical system perspective poses several challenges. In particular, it raises the question of how musical interaction can be described in a quantitative manner. If it is no longer valid to divide the system as a whole into its individual components so as to manage its complexity, how can we explore the dynamics of joint music making in real life? Taking a coordination dynamics perspective, we advocate a solution that places an understanding of the structuring principles of the behavior of dynamical systems at the root of this endeavor. The key to this understanding can be found in the patterns of recurrence that are a fundamental property of the behavior of dynamical systems.

Traditionally, the search for recurrent patterns in time series relies on linear methods such as auto- or cross-correlation, autoregressive models, and power spectral analysis, typically applied to a single time series such as measures of position or force. Musical time-series data such as audio or movement data, however, are often non-stationary and nonlinear, involving complex and diverse patterns that repeat irregularly at different locations or on different time scales (Demos et al., 2018, p. 246). Linear methods fall short in reliably capturing recurrence in these kinds of data, particularly at the level of the system. In the domain of DST, mathematical methods have been developed to compute nonlinear recurrence at the system level. In this domain, the dynamics at the system level is defined as a trajectory in a multidimensional (Euclidean) *phase space*, whose dimensions (axes) are defined by all the variables that exert an influence on the system’s current state and its evolution over time. It has been argued that, if chosen carefully, we could use a single observed time series to reliably reconstruct the dynamics of the system as a whole (cf. time-delay embedding method; Takens, 1981). By doing this we can compute recurrences of the system’s trajectory in the reconstructed phase space, which are foundational to the understanding of the dynamics of the system. The *recurrence plot* (RP) is a commonly used tool for visualizing recurrences in phase space, allowing the dynamics of the system to be described (Eckmann, 1987). Furthermore, the features of recurrence can be quantified using recurrence quantification analysis (RQA) (Webber & Zbilut, 2005).

*Phase space reconstruction*, the use of RPs, and RQA have received increasing interest in the domain of music performance and interaction (Demos et al., 2018; Dahl & Sioros, 2018; Ravignani, 2017; Varni et al., 2012). Demos and co-workers (2018), for example, carried out a rigorous study to find out if there were systematic relationships between the trombonists’ body sway and their musical phrasing. Here, body sway was considered from the perspective of its reconstructed trajectory in phase space and, in particular, the rate and stability of the recurrence of that trajectory. Results showed that the performance of musical phrases corresponded systematically with these recurrence metrics of the performers’ postural sway. Notably, this focus on recurrence metrics in phase space allowed them to pinpoint the relationship between bodily coordination and musical structure at the level of the organization of the system rather than at the more superficial level of spatiotemporal bodily gestures. The use of nonlinear methods to study interactions in music making—for example, those such as detrended fluctuation analysis (Hennig, 2014) and cross-wavelet spectral analysis (Walton et al., 2015)—has great potential. In particular, such methods enable experimental paradigms used for investigating musical variables in isolation, to be complemented by the quantitative study of interactions in music making and learning in more ecologically valid contexts. They also provide an overarching systems framework, within which behavioral and neuronal dynamics can be studied, linked with other methods of assessing the quality and meaning of musical interactions for individuals. This is not to say that traditional, linear methods are invalid or that they should be replaced by nonlinear methods. It is important to be aware, however, that when linear methods are applied to a real-life system that is nonlinear, the model of the system being used is inevitably approximate.

## The Dance of Individuality and Collectivity

One important advantage of the preliminary insights introduced here is that they permit explicit research questions to be tested. For example, in relation to music, does the co-generation and collective development of coordinated behavior into coherent spatiotemporal patterns rely on principles of self-organization? Here, the focus on interpersonal coordinated behavior may be understood in terms of large-scale systems in which biological (joints, muscles, neurons, other people, etc.) and non-biological elements (musical instruments, technologies, etc.) form a fluidly structured unit that sustains itself through its self-regulatory mechanisms. One way of beginning to answer this, and other similar questions, is to analyze the behavioral properties of the network with particular regard to the dynamics of stabilities and intermittent instabilities. Because DST explains the collective interaction of the system’s components as being directed toward stable attractor states, this stability may be jeopardized when the conditions that keep the system functional change. This may lead to critical fluctuations of the order parameter, and eventually to an abrupt transition to another attractor state. For open non-equilibrium systems, fluctuations and instabilities are fundamental to understanding how they (re)organize and evolve. By operating between order and disorder, systems can adapt flexibly to changing conditions, and may be triggered to evolve qualitatively new forms of organization. In the words of Waldrop this “edge of chaos [is] the one place where a complex system can be spontaneous, adaptive, and alive” (Waldrop, 1993, p. 12).

Arguably, this approach echoes earlier accounts of physico-chemical and biological evolution positing *free energy*,^4^ rather than the accumulation and preservation of *information*, as the driving force behind evolutionary processes (see Black, 1978; Lotka, 1922,^1956^). Yet much current research in cognitive, computational, and theoretical neuroscience places considerable emphasis on the *Bayesian brain hypothesis* and related principles of *predictive coding* and *free energy minimization* (see Clark, 2013; Friston, 2010, 2012). On this view, human beings can act upon their ability to infer the cause of sensory data and predict future states so as to adapt themselves to changing worldly conditions; according to Pezzulo and colleagues (2015) they can thus “resist the dispersive effects of external forces” (p. 18). Free energy and the experience of surprise might be seen as negatives, to be avoided if optimal functioning is to be achieved. On the basis of research on open non-equilibrium systems (e.g., Black, 1978; Kelso, 1995), however, a consideration of free energy as a fundamental principle driving the evolution of open, dynamical systems, may emerge. The study of musical participation is an ideal context in which to explore the dynamics of prediction and surprise.

Historically, this has been a central topic of investigation for researchers interested in understanding musical pleasure and emotion (Cheung et al., 2019; Gebauer et al., 2012; Huron, 2006; Meyer, 1956; Vuust et al., 2018). It can be argued that joint musicking is particularly engaging, expressive, and alive because of the subtle nuances and variations of musical parameters such as timing, note duration, intonation, and dynamics. New musical coordination patterns and subjectively felt qualities emerge in joint musicking through variation, surprise, and ambiguity, as do other facets of music making such as the relationships that unfold between musical instruments, body movements, and room acoustics. Shifts of state resulting from exploration, variability, and fluctuation take place on a continuum from the micro- to the macro-level. They may range from subtle changes of quality, as in the case of musical groove (Roholt, 2013), to the more marked changes in sound making and coordination in musical improvisation (Borgo, 2005; Corbett, 2016) and global changes of style in the musical repertoire that is performed. On this last point, Byrne (2012) has argued for example that variability in room acoustics has played an important role in the history of Western music since the Middle Ages. The exploration of the possibilities afforded by the study of surprise and deviation can reveal a diversity of novel patterns and other highly rewarding characteristics of music. Thus, it may be suggested that art in general, and music making in particular, may benefit not only from the minimization of free energy, but also from recognizing that free energy can be a driving force of creation, adaptation, renewal, affect, and agency.

More generally, the study of interpersonal coordination in dialog offers a good example of how similar insights can be addressed empirically. Recent work by Fusaroli and colleagues (2014), among others, seeks to provide explanations of a collective phenomenon—how dyads interact meaningfully—by exploring the patterns of self-regulation displayed by each lower level component (e.g., the listener and the speaker). As Cummins (2014) comments, this contribution

emphasizes the intertwining of the movements of participants, leading to dimensional reduction, so that two interacting persons become, temporarily, a simpler collective entity than the two persons considered as a mere conjunction of individuals. It acknowledges both synchronized and complementary actions as they contribute to this simplification, and it emphasizes the manner in which shared understanding of task constraints leads to stability of patterning in time.

A similarly synergistic approach has been recently applied to the study of expressive violin-piano duet performance by Coorevits and co-workers (2019). On the initial premise that tempo is an important variable for a system, they explored the effects of gradual tempo changes on articulatory patterns in the violinists’ body movements (head, right wrist, and body downward force) and showed that continuous changes in performance tempo led to transitions from one order to another characterized by qualitatively distinct bodily articulation patterns. Remarkably, such transitions occurred at tempi corresponding to the boundaries between those typically categorized in Western classical music as *largo* and *adagio, adagio* and *andante, andante* and *moderato*, and *allegro* and *presto.* This suggests that these categorizations may be rooted in the histories of structural couplings between the corporeal possibilities of agents and their social, cultural, and historical environment (see Thompson, 2007).

The apparent links between sedimented cultural contingencies, bodily proclivities, and self-organization raise an important challenge to the framework that we propose: exploring the role of subjectivity. Indeed, a system describing musical participation involves a number of living organisms, with their bubbles of experience, drives, needs, and histories of coupling with their material and social environment. In short, the participatory system needs to be described *functionally*, in terms of observable interactions between its components (local dynamics) and between its components and the whole system (global dynamics), as well as *phenomenologically*, in terms of the association between the local and global dynamics of the system with participants’ reported experiences of music making.

While the integration of these aspects poses important challenges, it can also open up novel lines of enquiry. New models could be used to predict how musicians playing together will respond collectively to particular perturbations by reorganizing different parameters of their behavior, for example by initiating novel musical actions that depend structurally on material previously heard or played (e.g., variations). DST offers ways for aspects of musical behavior to be measured at both individual and collective levels; these can be then integrated with qualitative accounts of musicians’ experiences obtained from individual and focus group interviews. The systematic comparison of musicians’ insights into experience, action, individuality, and collectivity would enable them to be studied both locally and globally, to reveal each musician’s functional contribution to the realization of a specific state of the system, and their perspective on their own experience of the performative, emotional, social, and creative dimensions of the shared musical event (see also Schiavio & Benedek, 2020). Here, behavioral and experiential degrees of freedom could be associated with different aspects of individual and collective experience, producing explanatory models combining first-, second- and third-person^5^ accounts (Gesbert et al., *under review*). Individual semi-structured interviews or self-assessment instruments could be used to obtain first-person data. Focus groups could be used to provide second-person data for the purpose of integrating internal and external components of the system; these would be particularly effective if members were to view and discuss a stimulus such as a video-recording of one of their performances. Finally, at the third-person level, mathematical models could be used to calculate the entropy levels of the system and its components in the light of its openness and adaptivity to external perturbation. The possibilities for integrated research, as described here, align closely with the characterization of musical participation as the open, non-equilibrium, dynamical system we have proposed. Openness, transitory phases, and changes over time can be addressed both qualitatively and quantitatively, both individually and collectively. Identifying this complex network of possibilities for research and analysis could inspire richer understandings of the complex dynamics of musical participation and its underlying mechanisms.

## Conclusion

People engage in a multitude of joint musical interactions, ranging from ritualistic singing in small groups, to mass dance events such as Tomorrowland.^6^ What is truly remarkable about these interactions is the way people organize what appear to be dissociated behaviors, emotional responses, and brain dynamics into strongly coupled patterns of activity, and that these patterns might be captured, at least in part, using certain strategies (e.g., Acquadro et al., 2016). Although the perspective we propose is still highly speculative, the insight that musical participation can best be viewed through the lens of self-organization may have interesting methodological implications for empirically informed musicology, opening up promising avenues for theory and research. Existing methods based on finding average differences and linear correlations are necessarily limited in capturing the spatiotemporal complexities, inter-subjective diversity, and temporal changes intrinsic to musical interactions. This has arguably impeded the study of musical interactions in more ecologically valid environments. Taking a DST-informed perspective on musical participation can help overcome these limitations by exploring the generic structuring principles that underlie observed behavioral complexity in ecological contexts. Here, concepts such as *attractor state, order parameter*, and *open non-equilibrium system*, can be adopted to better address the underlying dynamics at the basis of musical participation. As previously mentioned, an attractor state may be understood as the novel state to which the system is drawn; an order parameter is conceived of, rather, as a measure of the degree of order involved in the various transitions of the system; open non-equilibrium systems are defined in terms of their constantly shifting nature, subject to both endogenous and exogenous influences. Here the focus on the outcome of musical interaction is traded for the study of the organizational and structural principles that drive and constrain this activity as an evolving, synergistic, dynamical network. Research informed by DST can be used to build on previous work that places emphasis on ensuring ecological validity in empirical investigations by offering diverse heuristics. Here, the unit of analysis shifts from the individual to the collective, such that they can both be taken into account. As is the case for all open, non-equilibrium systems, the dynamical interplay of regulatory and perturbatory forces, of stability and fluctuation, of predictability and surprise, lie at the core of these principles. They should be the specific focus of future research aiming to increase our knowledge of how musically meaningful interactions (among musicians, listeners, etc.) unfold and develop, and how musical skills emerge from the integration of a multiplicity of forces that shape individual and collective learning trajectories.

## Notes

1. By *motor plans* we mean a set of organizational processes involving the selection and preparation of a given movement to be performed voluntarily (see e.g., Gallistel, 1980).

2. By *motor repertoire* we mean the spectrum of goal-directed behavioral configurations that can be enacted by an agent or, in other words, the vocabulary of action constituting their motor expertise. For example, the motor repertoire exhibited by an expert musician is very different from that of an expert tennis player (see Calvo-Merino et al., 2005; Gallese, 2007).

3. As Di Paolo and De Jaegher (2012) comment: “the brain is potentially less involved in reconstructing or computing the ‘mental state’ of others based on social stimuli and more involved in participating in a dynamical process outside its full control, thus inviting explanatory strategies in terms of dynamical concepts such as synergies, coordination phase attraction, (meta)stability, structural stability, transients, and stationarity.”

4. According to Friston and colleagues (2012), “free energy” is “an information-theoretic [. . .] quantity [that] bounds surprise, conceived as the difference between an organism’s predictions about its sensory inputs (embodied in its models of the world) and the sensations it actually encounters.”

5. A description of the tension between first- and third-person perspectives in musical and scientific literature can be found in Leman’s *Embodied music cognition and mediation technology* (2007). Here, Leman also suggests that interaction is central to second-person approaches: “second-person descriptions are used to show, express, and articulate the private experience from one subject to another. They imply a ‘me-to-you’ relationship” (p. 82). As this level of analysis comprises “non-verbal articulations as well as verbal descriptions” (p. 82), models informed by DST could build on such an integrative account to examine the behavioral, linguistics, and emotional features of the ongoing interactive process in more detail (see also de Bruin et al., 2012; Gnisci et al., 2008).

6. An electronic dance music festival held in Belgium.
